# Effect of body mass index on serum urate and renal uric acid handling responses to an oral inosine load: experimental intervention study in healthy volunteers

**DOI:** 10.1186/s13075-020-02357-y

**Published:** 2020-11-04

**Authors:** Nicola Dalbeth, Jordyn Allan, Gregory D. Gamble, Anne Horne, Owen M. Woodward, Lisa K. Stamp, Tony R. Merriman

**Affiliations:** 1 Department of Medicine, Faculty of Medical and Health Sciences, University of Auckland, 85 Park Rd, Grafton, Auckland, 1023 New Zealand; 2 Department of Physiology, University of Maryland School of Medicine, Baltimore, USA; 3 Department of Medicine, University of Otago, Christchurch, New Zealand; 4 Department of Biochemistry, University of Otago, Dunedin, New Zealand

**Keywords:** Urate, Gout, Body mass index, Purine, Inosine

## Abstract

**Background:**

High body mass index (BMI) is strongly associated with hyperuricaemia. It is unknown whether overweight and obesity influences serum urate primarily through increased urate production or reduced renal clearance of uric acid. The aim of this study was to determine the influence of BMI on the response to inosine, a purine nucleoside that functions as an intermediate in the purine salvage and degradation pathways.

**Methods:**

Following an overnight fast, 100 healthy participants without gout attended a study visit. Blood and urine samples were taken prior to and over 180 min after 1.5 g oral inosine. Serum urate and fractional excretion of uric acid (FEUA) were analysed according to high BMI (≥ 25 kg/m^2^) and low/normal BMI (< 25 kg/m^2^) groups, and according to BMI as a continuous variable.

**Results:**

Participants in the high BMI group (*n* = 52, mean BMI 30.8 kg/m^2^) had higher serum urate concentrations at baseline (*P* = 0.002) compared to those with low/normal BMI (mean BMI 21.8 kg/m^2^). However, the high BMI group had a smaller increase in serum urate following the inosine load (*P* = 0.0012). The two BMI groups had a similar FEUA at baseline (*P* = 0.995), but those in the high BMI group had a smaller increase in FEUA following the inosine (*P* = 0.0003). Similar findings were observed when analysing BMI as a continuous variable. Those with high BMI had a smaller increase in FEUA per increase in serum urate, compared to those with low BMI (*P* = 0.005).

**Conclusions:**

In a fasting state, people with high BMI have elevated serum urate levels but similar FEUA values compared with those with low/normal BMI. Following a purine load, those with high BMI have an attenuated renal excretion of uric acid. These data, using an experimental method to dynamically assess human urate handling, suggest that people with high BMI have a higher renal capacity for uric acid reabsorption when fasted and following a dietary purine intake have reduced renal clearance.

**Trial registration:**

Australia and New Zealand Clinical Trials Registry, ACTRN12615001302549, date of registration 30 November 2015.

## Background

High body mass index (BMI) is an important risk factor for hyperuricaemia. A cross-sectional analysis of National Health and Nutrition Examination Survey (NHANES) III estimated that the population attributable risk of BMI > 25 kg/m^2^ for hyperuricaemia is 40% [1]. In longitudinal studies, higher BMI is a risk factor for hyperuricaemia; in Japanese men, the hazard ratio for incident hyperuricaemia over 8 years was 1.19 for a BMI increase of 2.64 kg/m^2^ [2]. Weight loss leads to modest reductions in serum urate [3]. It is unknown whether overweight and obesity influences serum urate primarily through increased urate production or reduced renal clearance of uric acid.

Inosine is a purine nucleoside that is metabolised in vivo from adenosine monophosphate (AMP) as part of the purine salvage metabolic pathway. Inosine is degraded to hypoxanthine, which is, in turn, metabolised to xanthine and then urate in the purine degradation pathway. Administration of inosine increases serum urate concentrations [4]. Thus, oral administration of a fixed dose of inosine allows analysis of the acute effects of a standardised purine load on both serum urate concentrations and renal uric acid handling. The aim of this study was to determine the influence of body mass index on the response to an oral inosine load.

## Participants and methods

This was an experimental intervention study designed to identify factors that influence serum urate and fractional excretion of uric acid (FEUA) responses to inosine. The primary aim of the study was to determine the influence of genetic variants on inosine-induced hyperuricaemia in healthy adult volunteers, and these results have been reported in full [5]. The study protocol also pre-specified analysis of the effects of BMI on serum urate and FEUA responses to inosine. The primary endpoint of the study was change in serum urate over 180 min after inosine ingestion. The secondary endpoint was change in fractional excretion of uric acid over 180 min after inosine ingestion. The study protocol was modified from previous experimental intervention studies investigating the acute effects of skim milk, fructose, and frusemide on urate concentrations and renal excretion of uric acid in healthy volunteers [6–9].

### Participants

One hundred healthy participants were recruited by public advertising. Indigenous New Zealanders (Māori) and Pacific people living in Aotearoa/New Zealand have a very high prevalence of hyperuricaemia and gout [10], and for this reason, the sampling framework in the study protocol specified recruitment of 50 participants of Māori or Pacific ethnicity, and 50 participants of New Zealand European ethnicity. Inclusion criteria were as follows: the ability to provide written informed consent, and estimated glomerular filtration rate (eGFR) > 60 mL/min/1.73 m^2^. Exclusion criteria were as follows: ethnicity other than Māori, Pacific people, or New Zealand European; first degree relative of another study participant; history of gout; history of kidney stones; history of diabetes mellitus; diuretic use; or urine pH ≤ 5.0.

Potential participants attended a screening visit where a general health questionnaire was completed and baseline measurements (including weight and height) and physical examination were performed. Screening blood tests were also obtained. Screening visits were completed for 110 participants. Seven prospective participants were excluded following the screening visit (four participants were of ethnicity other than Māori, Pacific people, or New Zealand European; two participants who were first degree relative of another study participant; one due to health problems). Three participants who fulfilled criteria for the study following screening did not proceed to the study visit. All visits took place at a clinical research facility in a tertiary medical centre. The study was approved by the New Zealand Health and Disability Ethics Committee (MEC/05/10/130), and each participant gave written informed consent. The study was registered by the Australian Clinical Trials Registry (ACTRN12615001302549).

### Protocol

The study visit occurred within 2 weeks of the screening visit. At the study visit, a venous catheter was inserted for blood collection. Having fasted overnight, the participants took three 500-mg inosine tablets (Source Naturals, Scotts Valley, CA) orally over a 5-min period between 0800 and 0930. Blood was obtained for urate, glucose, and creatinine prior to ingestion, and then 15 min, 30 min, 60 min, 120 min, and 180 min after ingestion. Urine was obtained for urate and creatinine prior to ingestion, and then 30 min, 60 min, 120 min, and 180 min after ingestion. Urine volume was measured at each time point. Drinking water was also provided at each time point (30, 60, 120, and 180 min) to a volume equivalent to the collected urine volume. A light meal was provided at the end of the study following collection of all samples and removal of the venous catheter.

Each inosine tablet contained 500 mg inosine, 30 mg calcium, plus dibasic calcium phosphate, sorbitol, stearic acid, modified cellulose gum, and magnesium stearate. The dose of 1.5 g is consistent with doses used in other clinical trials of inosine [4, 11] and is within the recommended daily dose range when used as an over the counter supplement. In a long-term study of inosine in Parkinson’s disease, a mean (SD) urate increase of 0.18 (0.07) mmol/L was observed with a mean dose of 1.53 g inosine [4].

### Laboratory testing

Serum and urine chemistry was tested using the Roche Modular P (Hitachi) analyser. The FEUA was calculated; this is the ratio between the renal clearance of uric acid and the renal clearance of creatinine, expressed as a percentage.

### Statistical analysis

The analysis plan for this study specified change in serum urate concentration as the primary endpoint. The key secondary endpoint was change in FEUA. Based on our prior study of fructose intake in healthy volunteers [9], the expected percentage of study participants with BMI of 25 kg/m^2^ or more was 59%; the study had 80% power at the 5% significance level for a two-tailed test assuming an 0.04 mmol/L SD of the change in serum urate as previously observed (PASS 16 Power Analysis and Sample Size Software (2018). NCSS, LLC. Kaysville, UT, USA, ncss.com/software/pass).

All analyses were conducted using SAS (v9.4 SAS Institute Inc., Cary, NC). Data are presented as mean (standard deviation (SD)) or *n* (%) for descriptive purposes; however, measures of effect are presented with the appropriate 95% confidence interval. Data were analysed according to high BMI (25 kg/m^2^ or more) and low/normal BMI (less than 25 kg/m^2^). The primary endpoint was change in serum urate, and secondary endpoint was change in FEUA. Data were analysed using a mixed models approach to repeated measures. Age, sex, and ethnicity were included in all models, unless stated. Significant group effects were explored using the method of Tukey. For change in serum urate, a mixed models analysis of covariance (ANCOVA) approach to repeated measures was used. For ANCOVA, the dependent variable was change from baseline, and baseline level was included as a covariate in the analysis. Interactions between absolute and change from baseline in both serum urate and FEUA for BMI as a continuous variable were visualised as effect plots using the PLM procedure of SAS with pre-specified adjustment for age, sex, and ethnicity and for change from baseline as appropriate baseline serum urate or FEUA. BMI was also analysed as a continuous variable for the two ethnicity groups, with adjustment for age and sex. Differences in participant characteristics between those in the high and low/normal BMI groups were analysed using *t* tests for normally distributed data and Fisher’s exact tests. Mixed model one-way analysis of variance (ANOVA) with Dunnett’s multiple comparison test was used to analyse changes from baseline over time in the entire group. No further adjustment for multiplicity was performed. *P* < 0.05 was considered significant, and all tests were two-tailed.

## Results

### Participant characteristics

Overall, the mean (SD) age of the participants was 29 (13) years, and 79 (79%) were female. There were 52 (52%) participants with BMI ≥ 25 kg/m^2^. For those in the high BMI group, the mean (SD) BMI was 30.8 (5.2) kg/m^2^, and for the low/normal BMI group was 21.8 (1.9) kg/m^2^. Age, sex, and serum creatinine were similar between the two BMI groups. The low/normal BMI group had more NZ European participants, and the high BMI group had more participants of Māori or Pacific ethnicity (Table 1). All subsequent mixed models analyses included ethnicity as a covariate.

**Table 1:** Baseline characteristics of study participants. Unless stated, data are presented as mean (SD). Unadjusted *P* values are shown

	BMI < 25 kg/m^**2**^	BMI ≥ 25 kg/m^**2**^	***P*** value
***n***	48	52	
**Age, years**	28.7 (11.5)	29.0 (14.5)	0.91
**Female sex,** ***n*** **(%)**	38 (79%)	41 (79%)	1.00
**Māori ethnicity,** ***n*** **(%)**	10 (21%)	11 (21%)	0.010
**Pacific ethnicity,** ***n*** **(%)**	6 (13%)	23 (44%)	
**NZ European ethnicity,** ***n*** **(%)**	32 (67%)	18 (35%)	
**Smoker,** ***n*** **(%)**	3 (6%)	3 (6%)	1.00
**Weight, kg**	64.5 (9.3)	88.9 (17.6)	2.8 × 10^−13^
**Height, cm**	171 (9)	169 (9)	0.29
**Body mass index, kg/m**^**2**^	21.8 (1.9)	30.8 (5.2)	9.9 × 10^−18^
**Serum creatinine, μmol/L**	70 (10)	71 (12)	0.59
**Serum glucose**	4.7 (0.4)	5.0 (0.4)	0.0003
**Serum urate, mmol/L**	0.27 (0.07)	0.32 (0.08)	0.0002
**Serum urate, mg/dL**	4.5 (1.2)	5.3 (1.3)	
**FEUA, %**	6.8 (2.4)	6.2 (2.4)	0.24
**FEUA in the lower tertile (≤ 5.22%),** ***n*** **(%)**	11	20	0.13
**FEUA in the highest tertile (≥ 6.81%),** ***n*** **(%)**	18	15	0.39

In the entire study population, the oral inosine load led to large increases in serum urate (mean increase 0.10 mmol/L; 1.6 mg/dL) and FEUA (mean increase 3.7%) over the 180-min study period (*P* < 0.0001 for both) (Supplementary Figure 1). No significant change was observed in the serum glucose over time (Supplementary Figure 1).

### Serum urate concentrations

**Fig. 1:**
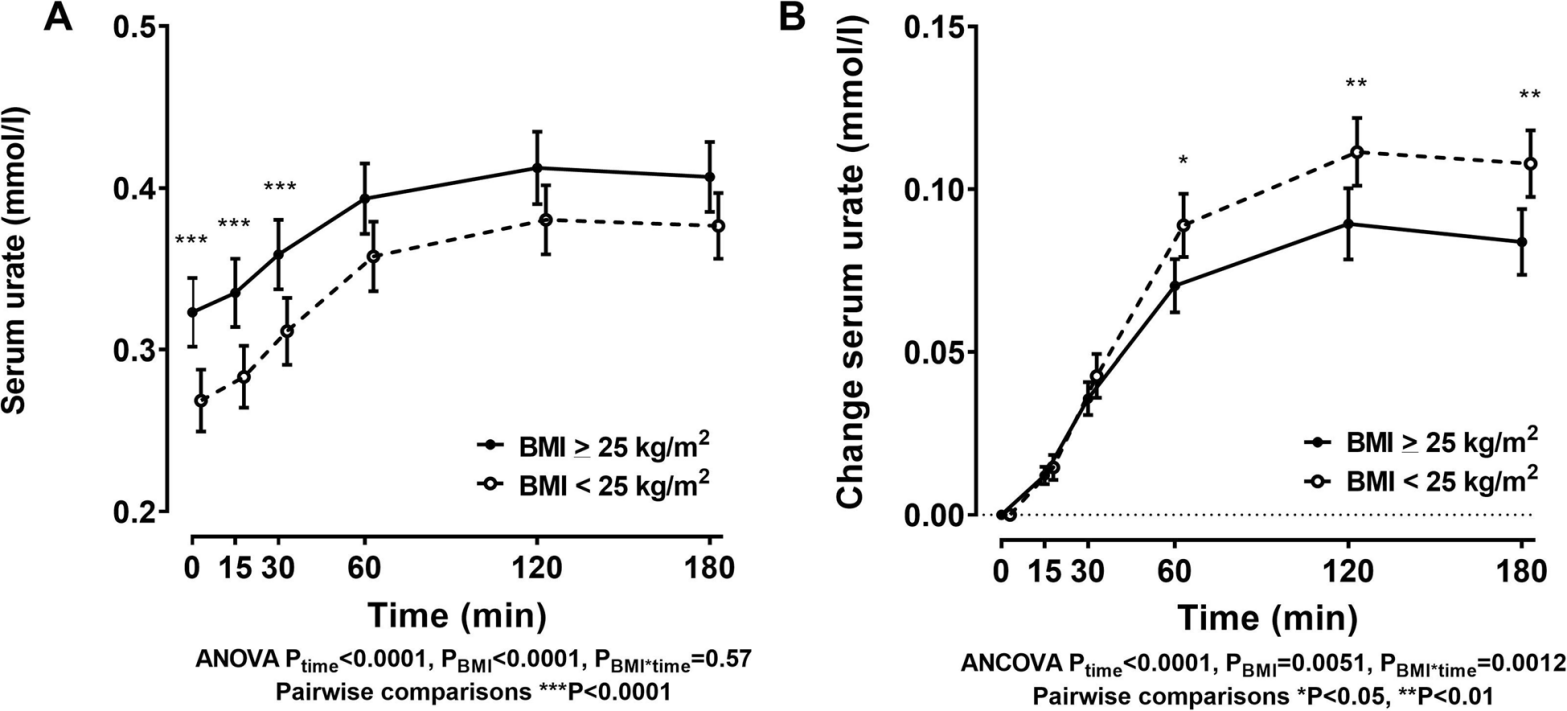
Serum urate following an oral inosine load according to BMI group. **a** Serum urate and **b** change in serum urate. Data are presented as unadjusted mean (95% CI). Age-, sex-, and ethnicity-adjusted *P* values are shown

Participants in the high BMI group had higher serum urate concentrations at baseline compared to those with low/normal BMI (Table 1 and Fig. 1a; age-, sex-, and ethnicity-adjusted *P* = 0.002). Serum urate increased in both groups following the inosine load (Fig. 1a, b). However, the increase in serum urate was greater in the low BMI group, compared to the high BMI group (Fig. 1b; ANCOVA *P*_BMI*time_ = 0.0012).

**Fig. 2:**
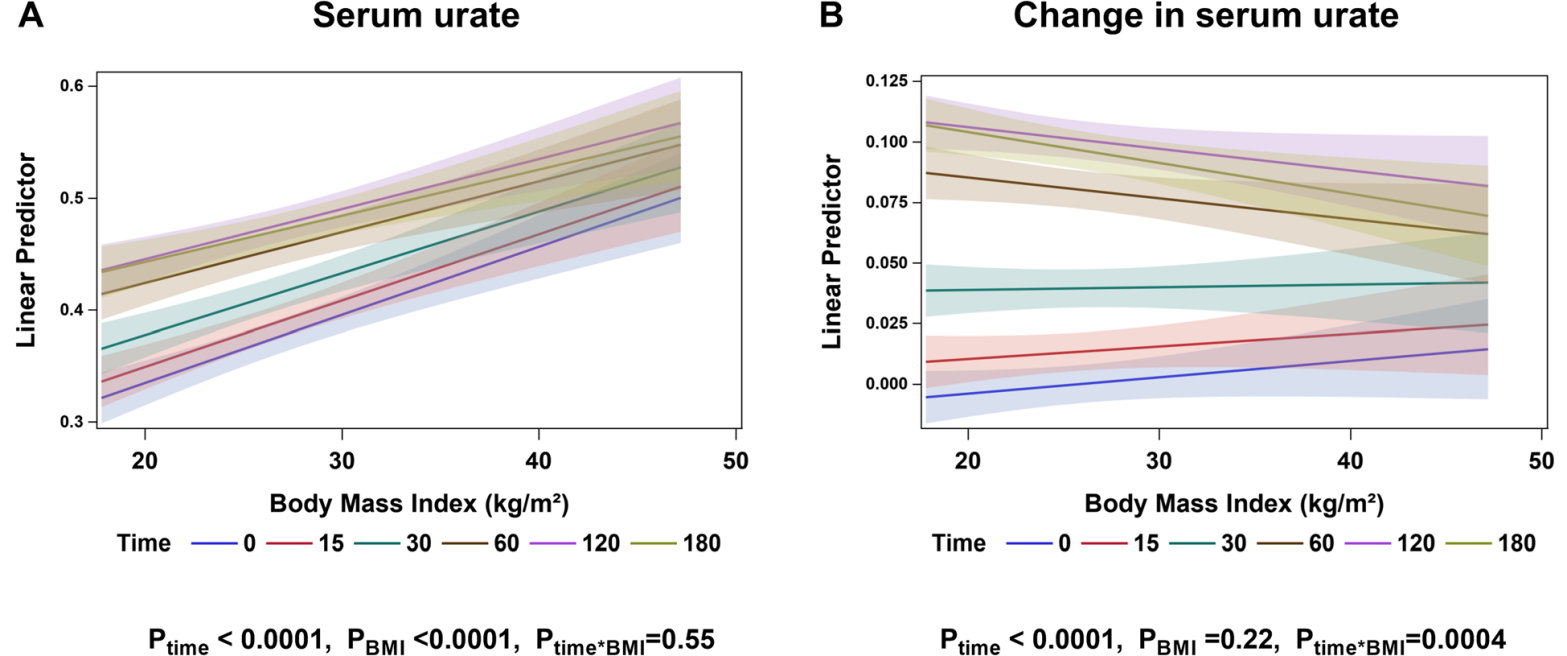
Serum urate at each time point according to BMI (kg/m^2^) as a continuous variable. **a** Serum urate (mmol/L) and **b** change in serum urate (mmol/L). Data are presented as adjusted mean (95% CI) for each time point. Age-, sex-, and ethnicity-adjusted *P* values are shown

When BMI was analysed as a continuous variable, increases in serum urate were observed across the BMI range following the inosine load (Fig. 2a). However, the increase in serum urate was greater at lower BMI values, compared to the higher BMI values (Fig. 2b; ANCOVA *P*_BMI*time_ = 0.0004). When data were separated by the ethnicity groups, this effect was most evident in participants of Māori or Pacific ethnicity (Supplementary Figure 2).

### Fractional excretion of urate

**Fig. 3:**
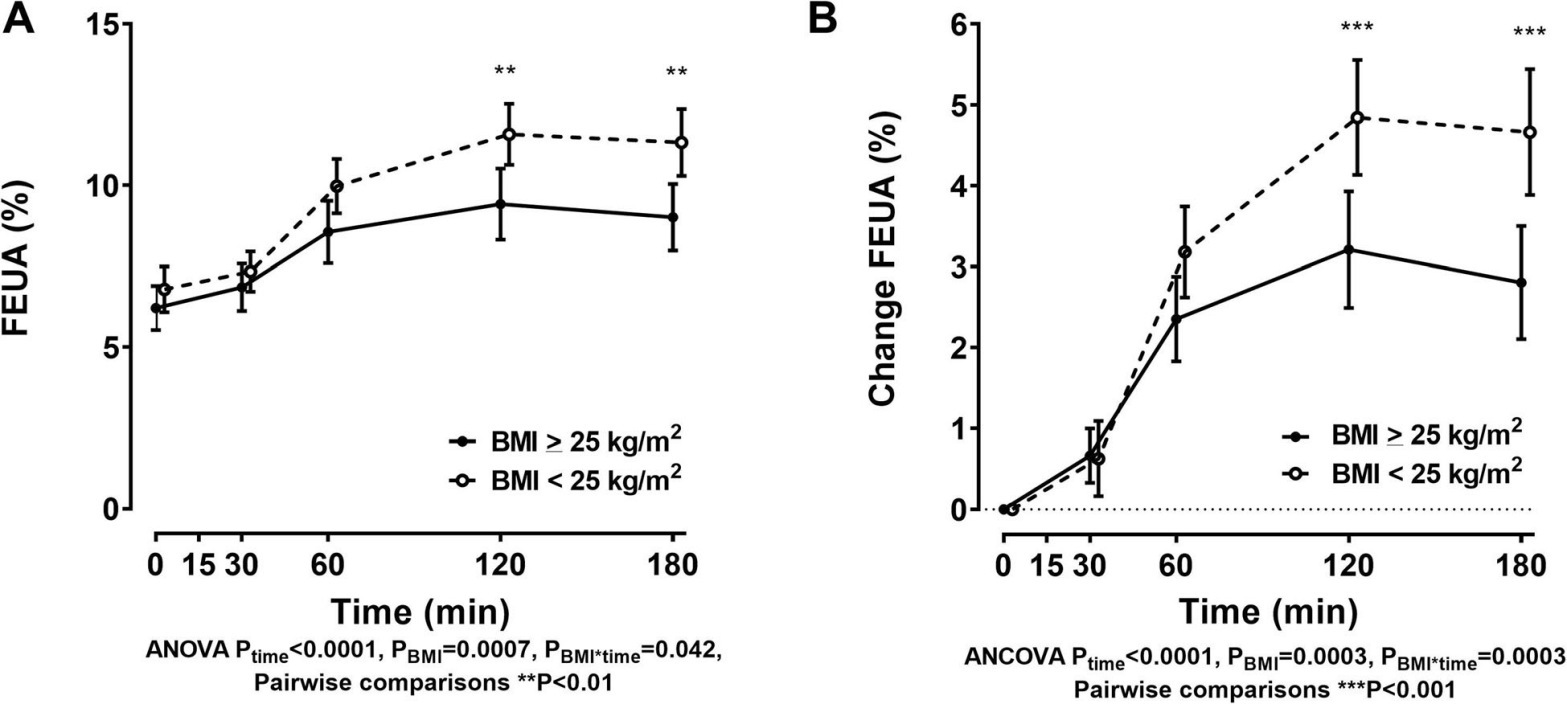
FEUA following an oral inosine load according to BMI group. **a** FEUA and **b** change in FEUA following an oral inosine load in different BMI groups. Data are presented as unadjusted mean (95% CI). Age-, sex-, and ethnicity-adjusted *P* values are shown

At baseline, participants in the high BMI group and low/normal BMI had similar FEUA values (Table 1 and Fig. 3a; age-, sex-, and ethnicity-adjusted *P* = 0.995). In addition, there was no difference between BMI groups based on the lowest or highest FEUA tertile at baseline (Table 1). FEUA increased in both groups following the inosine load (Fig. 3a, b). However, the increase in FEUA was greater in the low BMI group, compared to the high BMI group (Fig. 3b; ANCOVA *P*_BMI*time_ = 0.0003).

**Fig. 4:**
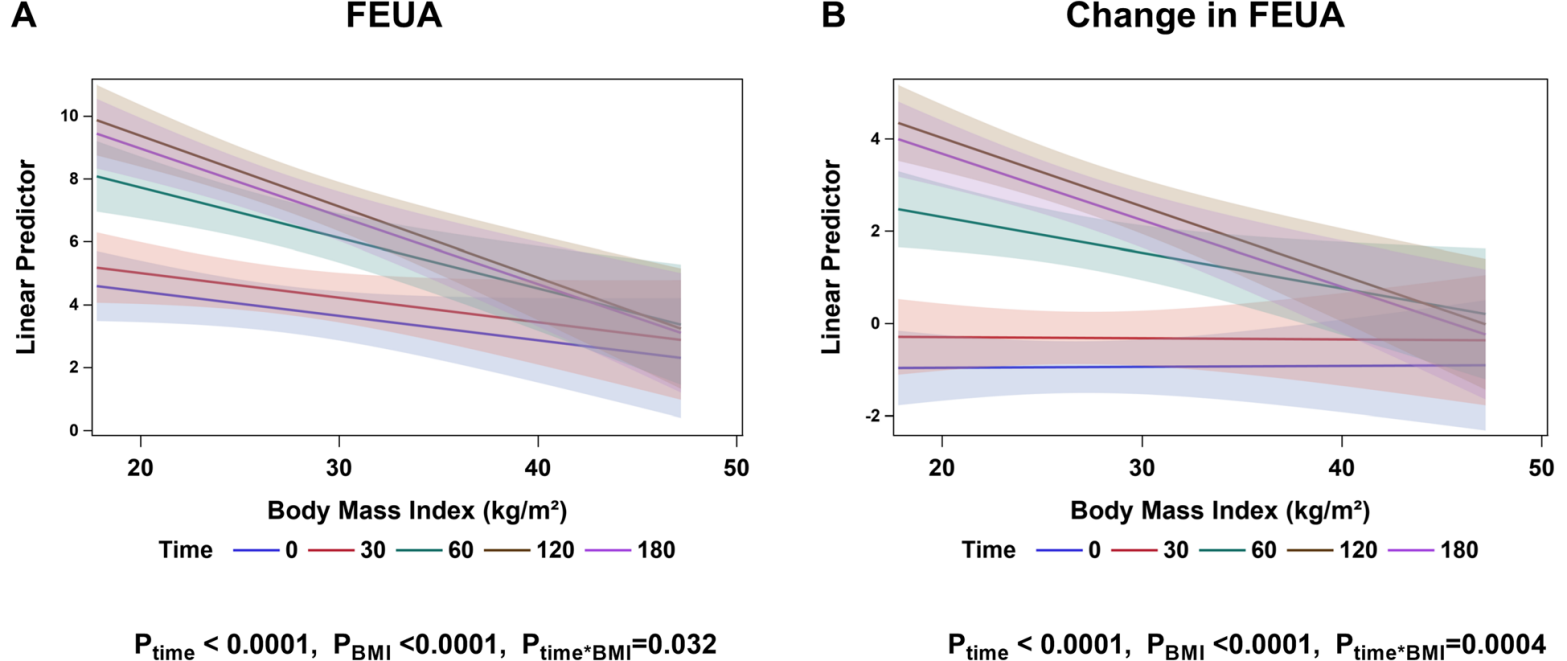
FEUA at each time point according to BMI (kg/m^2^) as a continuous variable. **a** FEUA (%) and **b** change in FEUA (%). Data are presented as adjusted mean (95% CI) FEUA (%). Age-, sex-, and ethnicity-adjusted *P* values are shown

When BMI was analysed as a continuous variable, increases in FEUA following the inosine load were most apparent in those with lower BMI values and were attenuated in those with higher BMI values (Fig. 4; ANCOVA *P*_BMI*time_ = 0.0004). Similar trends were observed in participants of Māori or Pacific ethnicity and in participants of New Zealand European ethnicity (Supplementary Figure 3).

### Serum glucose

Participants in the high BMI group had higher fasting glucose concentrations at baseline compared to those with low/normal BMI (Table 1 and Supplementary Figure 4; age-, sex-, and ethnicity-adjusted *P* = 0.008). Blood glucose remained higher in the high BMI group for most time points during the study (Supplementary Figure 4). In regression models of all participants that included BMI, no significant associations were observed between baseline blood glucose and FEUA or serum urate at any time points (*P* > 0.38 for all analyses). This was also the case for the same regression models that were restricted to participants of Māori or Pacific ethnicity (*P* > 0.45 for all analyses).

### Relationship between changes in serum urate and renal responses according to BMI group

**Fig. 5:**
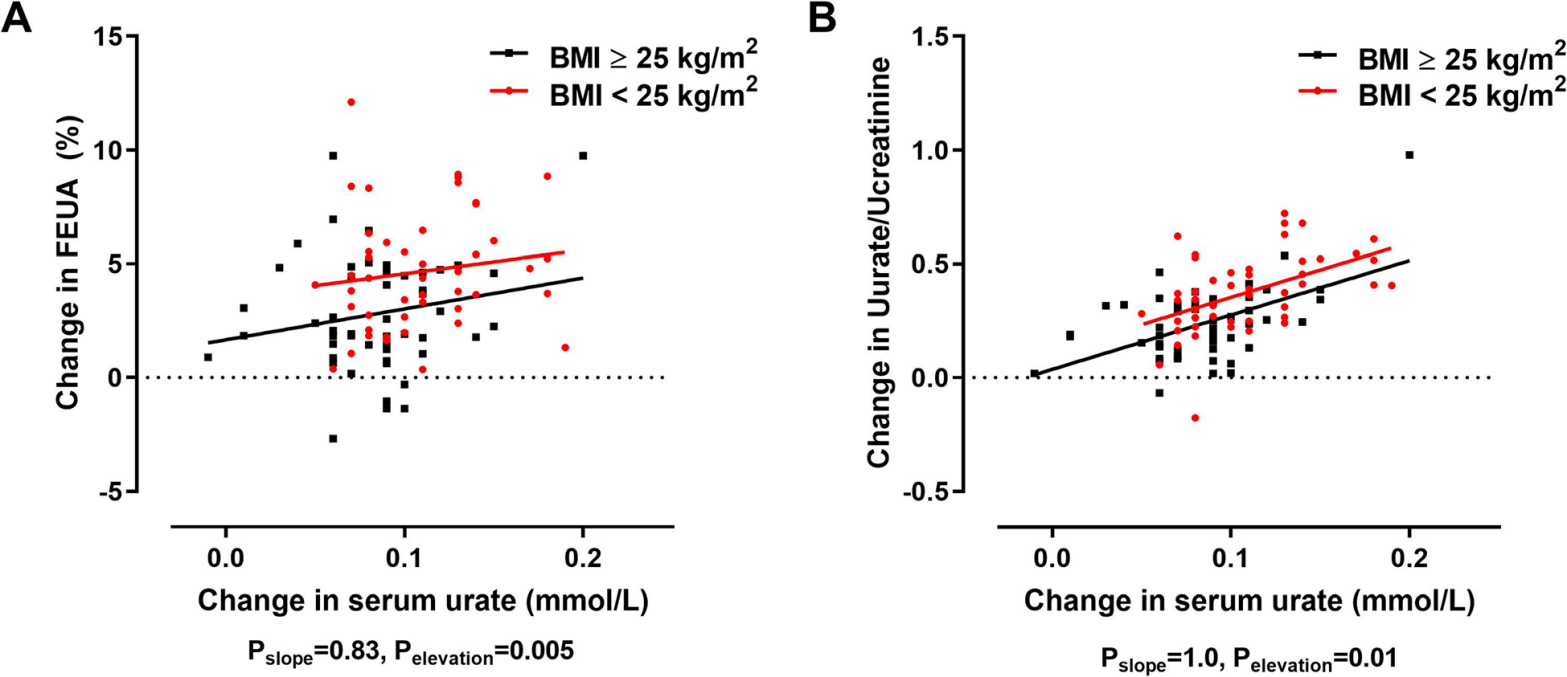
Relationship between changes in serum urate and renal responses according to BMI group. **a** Change from baseline in FEUA at 180 min. **b** Change from baseline in urinary uric acid/urinary creatinine ratio at 180 min

As FEUA is a function of serum urate, we examined whether the differences in the renal responses in the high BMI group were due to the differences in the serum urate response following the inosine load (Fig. 5). At the 180-min time point, the comparison of regression lines for change from baseline in FEUA vs change from baseline in serum urate plots demonstrated similar regression slopes (*P*_slope_ = 0.83), but a difference between the groups in elevation (*P*_elevation_ = 0.005) indicating that those with high BMI had a smaller increase in FEUA per increase in serum urate, compared to those with low BMI (Fig. 5a). Similar findings were observed when we examined the change in urinary uric acid/urinary creatinine; after 180 min, there were similar regression slopes (*P*_slope_ = 1.0), but a difference between the groups in elevation (*P*_elevation_ = 0.01) indicated that those with higher BMI had a smaller increase in urinary uric acid/urinary creatinine per increase in serum urate, compared to those with low BMI (Fig. 5b).

## Discussion

This study has demonstrated that people with overweight and obesity have higher serum urate concentration in a fasting state, but do not have an exaggerated hyperuricaemic response to a standardised oral purine load. Those with higher BMI have reduced renal excretion of uric acid following oral inosine loading, suggesting impaired renal clearance of uric acid following dietary purine intake in those with overweight and obesity.

An important finding of this study is that, despite higher baseline urate concentrations, those with overweight/obesity did not have an exaggerated serum urate response to the standard oral purine load. These findings do not indicate that there is greater transport of purines from the gut, nor that purines are preferentially degraded to urate in those with high BMI. On the contrary, the serum urate elevation in response to the inosine challenge was lower in those with high BMI; the cause of this finding is unclear but may be related to altered function of gut purine transporters or differences in the hepatic purine salvage and degradation pathways in those with high BMI.

At baseline, although fasting serum urate was elevated in the high BMI group, the FEUA was similar between the two groups. The increase in FEUA occurring after the oral challenge was smaller in those with high BMI. The similar FEUA at baseline despite the higher circulating urate concentrations, together with the attenuated FEUA response following the purine challenge, may, in turn, contribute to prolonged, elevated serum urate levels.

The FEUA findings align with our previous study examining the effects of BMI on serum urate and FEUA following an oral fructose load in a different group of participants [9]. In the fructose loading study, baseline serum urate levels were also elevated in the high BMI group, with similar baseline FEUA values compared with the low/normal BMI group. Studies of urate transport indicate that the machinery of transporters is essentially at capacity at baseline and that increasing FEUA with increasing load represents “spilling”, with the amount of uric acid filtered saturating reabsorptive transporters, so that extra uric acid is excreted [5]. Collectively, these observations suggest that people with high BMI have a higher capacity for uric acid reabsorption at baseline.

The increases in FEUA occurring after both the oral fructose challenge [9] and the inosine challenge were smaller in those with high BMI, indicating that renal clearance of a urate load (irrespective of the cause of the acute load) is less efficient in those with high BMI. In humans, urate reabsorption likely represents a mechanism by which the kidney protects itself from crystal formation in the nephron [12]. Therefore, chronic elevation of serum urate may upregulate the baseline capacity for reabsorption, seemingly at odds with need to excrete acute urate loads, in order to protect from crystallisation.

The mechanism of altered renal uric acid handling is currently unknown. Moderately elevated blood glucose does associate with elevated serum urate concentrations [13]. However, although fasting serum glucose levels were higher in the high BMI group at baseline, we did not observe a significant association between baseline serum glucose and FEUA or serum urate throughout the study period that was independent of BMI, suggesting that elevated blood glucose does not explain the observed differences in FEUA responses following the inosine challenge.

Altered renal uric acid handling could also be related to higher circulating insulin levels in those with high BMI. Prior studies have reported a close relationship between insulin resistance and urinary uric acid clearance [14, 15], and that insulin sensitivity (assessed by euglycaemic clamp) is a strong predictor of serum urate levels [16]. More recent data suggest that insulin may alter renal urate transporters, particularly urate transporter 1 (URAT-1), the apical membrane transporter that regulates uric acid reabsorption from the urinary space in the proximal renal tubule [17]. In rodent models of obesity, renal tubular expression of URAT-1 is increased [18]. In insulin-deficient rodent models, renal tubular expression of URAT-1 is decreased, and insulin infusion increases URAT-1 expression [19]. In the kidney epithelial cell line NRK-52E, URAT-1 expression is directly induced by insulin [19]. It is also possible that other circulating factors or epigenetic changes influence renal urate transporter function according to BMI. The study protocol did not include measurement of insulin levels, so we cannot further dissect the influence of circulating insulin levels on the renal uric acid handling following the inosine load; examining the mechanisms of altered renal uric acid handling in the context of high BMI will be the focus of future work.

The major strength of the study design was the ability to analyse changes in serum urate and FEUA in a standardised protocol using a fixed purine load. Limitations of this study include the relatively short study period. This study was designed to examine the short-term effects of the inosine load on serum urate concentrations and FEUA, and it is possible that BMI may influence long-term serum urate responses to purine intake in a different manner. The study participants were healthy volunteers who did not have gout, so our findings may not be generalisable to people with gout. In a normal diet, purines are not consumed in isolation, and the study design did not allow analysis of the effects of other dietary factors such as fructose and dairy protein that may influence the serum urate and FEUA responses to purine intake [6, 7].

## Conclusions

In a fasting state, people with high BMI have elevated serum urate levels but similar FEUA values compared with those with low/normal BMI. Following a purine load, those with high BMI have an attenuated renal excretion of uric acid. These data, using an experimental method to dynamically assess human urate handling, suggest that people with high BMI have a higher renal capacity for uric acid reabsorption when fasted and following a dietary purine intake have reduced renal clearance.

## Supplementary Information

**Additional file 1: Supplementary Figure 1.** Serum urate and FEUA following an oral inosine load in all participants. A Serum urate, B. FEUA, and C. glucose. Data are presented as unadjusted mean (95% CI).

**Additional file 2: Supplementary Figure 2.** Serum urate at each time point according to BMI (kg/m^2^) as a continuous variable according to ethnicity groups. A. Serum urate (mmol/L) and B. Change in serum urate (mmol/L). Data are presented as adjusted mean (95% CI) for each time point. Age and sex -adjusted *P* values are shown.

**Additional file 3: Supplementary Figure 3.** FEUA at each time point according to BMI (kg/m^2^) as a continuous variable according to ethnicity groups. A. FEUA (%) and B. Change in FEUA (%). Data are presented as adjusted mean (95% CI) for each time point. Age and sex -adjusted P values are shown.

**Additional file 4: Supplementary Figure 4.** Serum glucose following an oral inosine load according to BMI group. A. Serum glucose and B. Change in serum glucose following an oral inosine load in different BMI groups. Data are presented as unadjusted mean (95% CI). Age, sex and ethnicity-adjusted P values are shown.

## Data Availability

Data are available on reasonable request.

## Abbreviations

AMP: Adenosine monophosphate
ANCOVA: Analysis of covariance
ANOVA: Analysis of variance
BMI: Body mass index
eGFR: Estimated glomerular filtration rate
FEUA: Fractional excretion of uric acid
NHANES: National Health and Nutrition Examination Survey
SD: Standard deviation
URAT-1: Urate transporter 1

## References

[1] Choi HK, McCormick N, Lu N, Rai SK, Yokose C, Zhang Y (2020). Population impact attributable to modifiable risk factors for hyperuricemia. Arthritis Rheumatol.

[2] Nakanishi N, Yoshida H, Nakamura K, Suzuki K, Tatara K (2001). Predictors for development of hyperuricemia: an 8-year longitudinal study in middle-aged Japanese men. Metabolism.

[3] Zhu Y, Zhang Y, Choi HK (2010). The serum urate-lowering impact of weight loss among men with a high cardiovascular risk profile: the Multiple Risk Factor Intervention Trial. Rheumatology (Oxford).

[4] Schwarzschild MA, Ascherio A, Beal MF, Cudkowicz ME, Curhan GC, Parkinson Study Group S-PDI (2014). Inosine to increase serum and cerebrospinal fluid urate in Parkinson disease: a randomized clinical trial. JAMA Neurol.

[5] Hoque KM, Dixon EE, Lewis RM, Allan J, Gamble GD, Phipps-Green AJ (2020). The ABCG2 Q141K hyperuricemia and gout associated variant illuminates the physiology of human urate excretion. Nat Commun.

[6] Dalbeth N, Gamble GD, Horne A, Pool B, Purvis L, House ME (2013). Population-specific influence of SLC2A9 genotype on the acute hyperuricaemic response to a fructose load. Ann Rheum Dis.

[7] Dalbeth N, Wong S, Gamble GD, Horne A, Mason B, Pool B (2010). Acute effect of milk on serum urate concentrations: a randomised controlled crossover trial. Ann Rheum Dis.

[8] Dalbeth N, Allan J, Gamble GD, Phipps-Green A, Flynn TJ, Mihov B (2017). Influence of genetic variants on renal uric acid handling in response to frusemide: an acute intervention study. RMD Open.

[9] Dalbeth N, Phipps-Green A, House ME, Gamble GD, Horne A, Stamp LK (2015). Body mass index modulates the relationship of sugar-sweetened beverage intake with serum urate concentrations and gout. Arthritis Res Ther.

[10] Winnard D, Wright C, Taylor WJ, Jackson G, Te Karu L, Gow PJ (2012). National prevalence of gout derived from administrative health data in Aotearoa New Zealand. Rheumatology (Oxford).

[11] Markowitz CE, Spitsin S, Zimmerman V, Jacobs D, Udupa JK, Hooper DC (2009). The treatment of multiple sclerosis with inosine. J Altern Complement Med.

[12] Matsuo H, Chiba T, Nagamori S, Nakayama A, Domoto H, Phetdee K (2008). Mutations in glucose transporter 9 gene SLC2A9 cause renal hypouricemia. Am J Hum Genet.

[13] Choi HK, Ford ES (2008). Haemoglobin A1c, fasting glucose, serum C-peptide and insulin resistance in relation to serum uric acid levels--the Third National Health and Nutrition Examination Survey. Rheumatology (Oxford).

[14] Facchini F, Chen YD, Hollenbeck CB, Reaven GM (1991). Relationship between resistance to insulin-mediated glucose uptake, urinary uric acid clearance, and plasma uric acid concentration. JAMA.

[15] Quinones Galvan A, Natali A, Baldi S, Frascerra S, Sanna G, Ciociaro D (1995). Effect of insulin on uric acid excretion in humans. Am J Phys.

[16] Vuorinen-Markkola H, Yki-Järvinen H (1994). Hyperuricemia and insulin resistance. J Clin Endocrinol Metab.

[17] Enomoto A, Kimura H, Chairoungdua A, Shigeta Y, Jutabha P, Cha SH (2002). Molecular identification of a renal urate anion exchanger that regulates blood urate levels. Nature.

[18] Doshi M, Takiue Y, Saito H, Hosoyamada M (2011). The increased protein level of URAT1 was observed in obesity/metabolic syndrome model mice. Nucleosides Nucleotides Nucleic Acids.

[19] Toyoki D, Shibata S, Kuribayashi-Okuma E, Xu N, Ishizawa K, Hosoyamada M (2017). Insulin stimulates uric acid reabsorption via regulating urate transporter 1 and ATP-binding cassette subfamily G member 2. Am J Physiol Renal Physiol.

