# Supplementary figures and images for: Effect of body mass index on serum urate and renal uric acid handling responses to an oral inosine load: experimental intervention study in healthy volunteers
Source: Arthritis Res Ther. 2020 Nov 4;22:259. doi: 10.1186/s13075-020-02357-y (PMC7641836; doi:10.1186/s13075-020-02357-y)

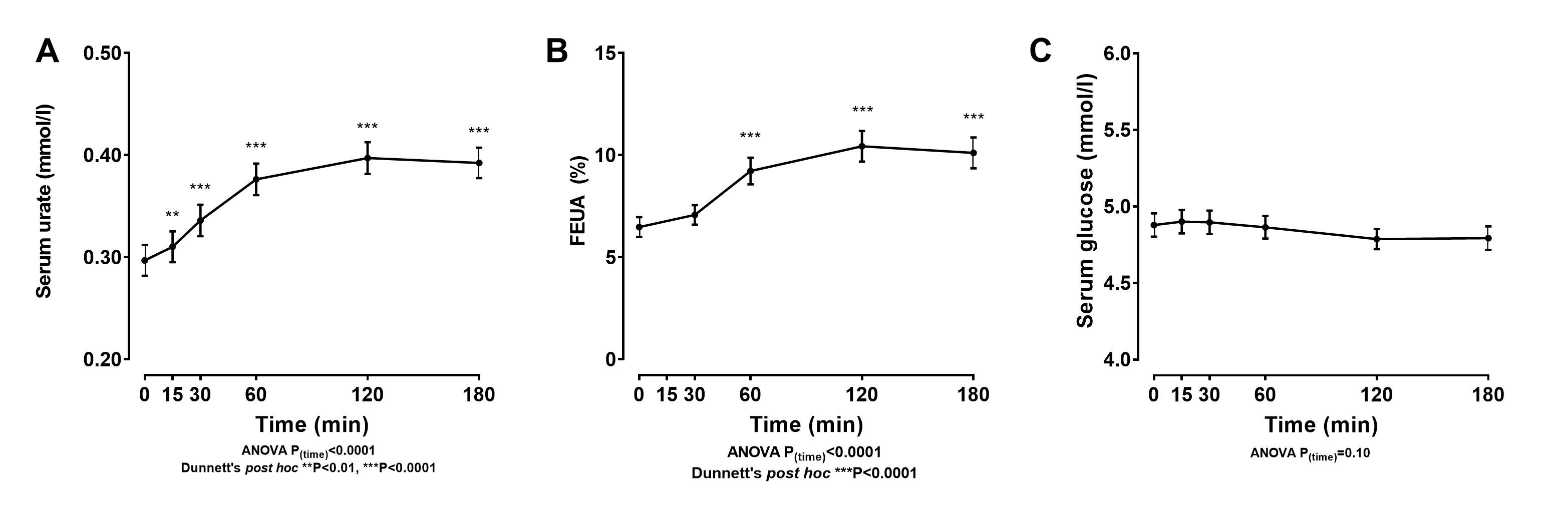

Supplement: Supplementary file 1 — Additional file 1: Supplementary Figure 1. Serum urate and FEUA following an oral inosine load in all participants. A Serum urate, B. FEUA, and C. glucose. Data are presented as unadjusted mean (95% CI). [file 13075_2020_2357_MOESM1_ESM.jpg]

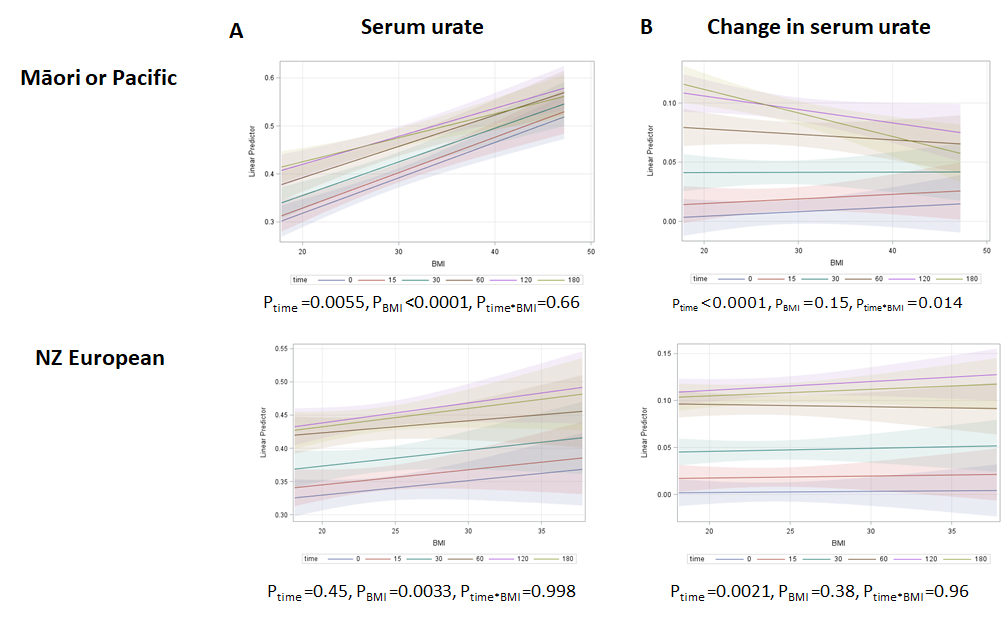

Supplement: Supplementary file 2 — Additional file 2: Supplementary Figure 2. Serum urate at each time point according to BMI (kg/m2) as a continuous variable according to ethnicity groups. A. Serum urate (mmol/L) and B. Change in serum urate (mmol/L). Data are presented as adjusted mean (95% CI) for each time point. Age and sex -adjusted P values are shown. [file 13075_2020_2357_MOESM2_ESM.jpg]

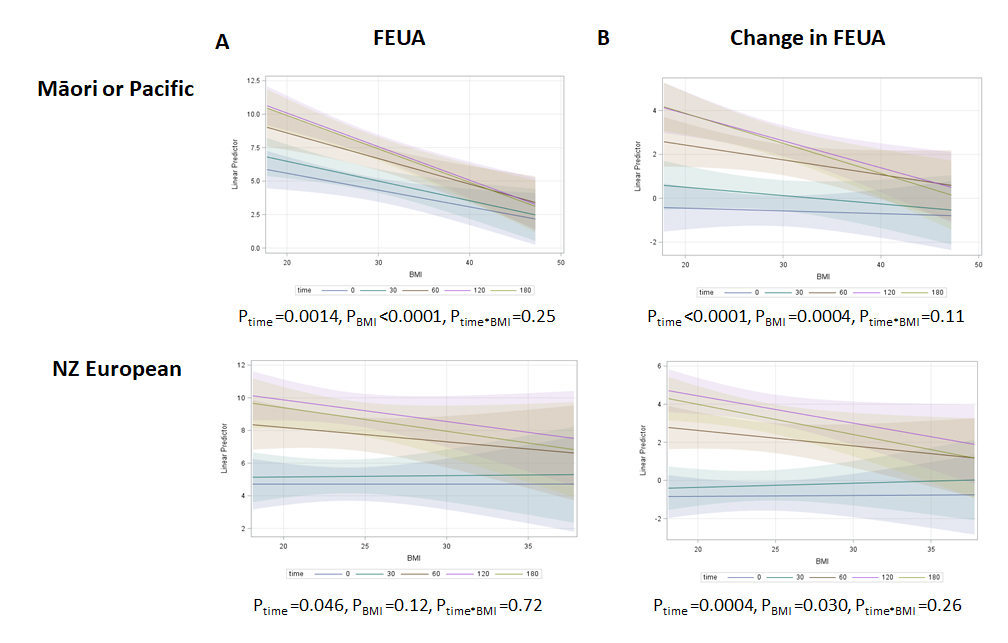

Supplement: Supplementary file 3 — Additional file 3: Supplementary Figure 3. FEUA at each time point according to BMI (kg/m2) as a continuous variable according to ethnicity groups. A. FEUA (%) and B. Change in FEUA (%). Data are presented as adjusted mean (95% CI) for each time point. Age and sex -adjusted P values are shown. [file 13075_2020_2357_MOESM3_ESM.jpg]

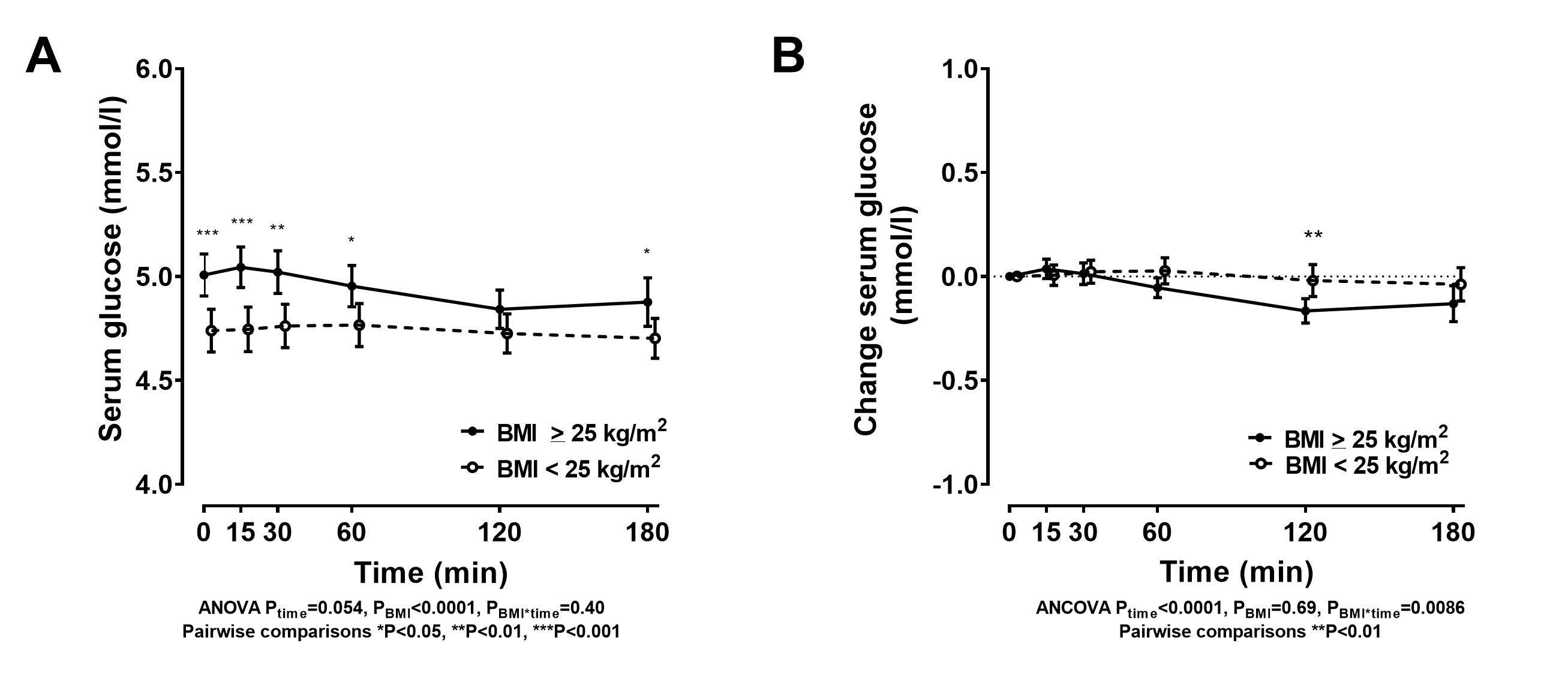

Supplement: Supplementary file 4 — Additional file 4: Supplementary Figure 4. Serum glucose following an oral inosine load according to BMI group. A. Serum glucose and B. Change in serum glucose following an oral inosine load in different BMI groups. Data are presented as unadjusted mean (95% CI). Age, sex and ethnicity-adjusted P values are shown. [file 13075_2020_2357_MOESM4_ESM.jpg]
